# Covalent bonds in positron dihalides†
†Electronic supplementary information (ESI) available: Details of the positron basis set construction, dissociation channels and thermodynamic cycles, a molecular orbital theory model of the positron bond, potential energy curves and densities not shown in the main document, electron binding energies, energy decomposition results and counterpoise correction tables. See DOI: 10.1039/c9sc04433g


**DOI:** 10.1039/c9sc04433g

**Published:** 2019-10-29

**Authors:** Félix Moncada, Laura Pedraza-González, Jorge Charry, Márcio T. do N. Varella, Andrés Reyes

**Affiliations:** a Department of Chemistry , Universidad Nacional de Colombia , Av. Cra 30 # 45-03 , Bogotá , Colombia . Email: areyesv@unal.edu.co; b Instituto de Física , Universidade de São Paulo , Rua do Matão 1731 , 05508-090 São Paulo , Brazil

## Abstract

We report a computational study on homo- and heteronuclear e^+^[X^–^Y^–^] compounds formed by two halide anions (X^–^, Y^–^ = F^–^, Cl^–^, Br^–^) and one positron. Our results indicate the formation of energetically stable positronic molecules in all cases. Analysis of the electron and positron densities points out that the formation of positron covalent bonds underlies the stabilization of the otherwise repelling dihalides, revealing that positronic bonding can reach far beyond the previously addressed e^+^[H^–^H^–^] molecule [J. Charry, M. T. do N. Varella and A. Reyes, *Angew. Chem. Int. Ed.*, 2018, **57**, 8859–8864.]. To a significant extent, the properties of the positron dihalides are similar to those of the purely electronic analogs, e^–^[A^+^B^+^], molecular cations with isoelectronic atomic cores (A^+^, B^+^ = Na^+^, K^+^, Rb^+^) bound by one electron. The positron bonds in the e^+^[X^–^Y^–^] complexes are however stronger than those in the isoelectronic e^–^[A^+^B^+^] counterparts, as the former have shorter bond lengths and higher bond energies. While an energy decomposition analysis points out that both electronic and positronic bonds essentially arise from electrostatic interactions, the more stable positron bonds are partly due to the higher polarizabilities of the dihalide anions, and partly to more significant contributions from correlation and relaxation effects.

## Abstract 2

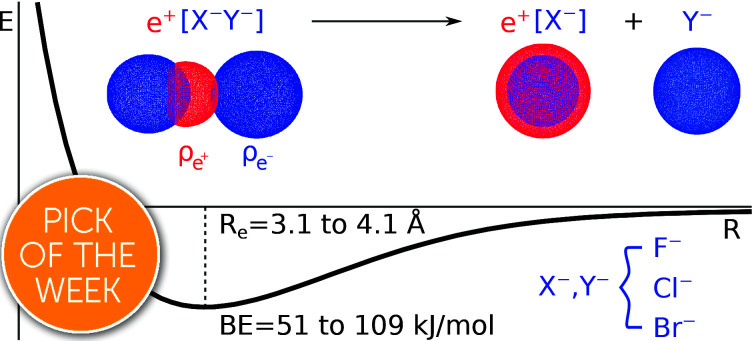
We report a computational study on homo- and heteronuclear e^+^[X^–^Y^–^] compounds formed by two halide anions (X^–^, Y^–^ = F^–^, Cl^–^, Br^–^) and one positron.

## 1 Introduction

Fundamental positron and positronium (Ps) chemical physics has long been a reality.^1^,^2^ Even a place for the Ps atom in the periodic table^3^ and term symbols for atomic and diatomic positronic species were proposed.^4^ For quite some time, however, the field was often plagued by a gap between the fascinating predictions of new species and phenomena, based on theory and numerical methods, and experimental realization. The situation drastically changed, for better, in more recent years. Unprecedented progress was made possible by the techniques to accumulate and manipulate positrons^5^ and Ps atoms^6^ at very low energies, and by Ps^–^ emission from metal surfaces.^7^ Among many other breakthroughs, one could mention the observation of an optically excited Ps^–^ resonance,^8^ Ps_2_ molecules,^9^,^10^ Ps-molecule transient states,^11^,^12^ and positronic molecules.^13^ Already in 2010, Gribakin *et al.*^14^ pointed out that about 60 positronic molecules were produced by low energy collisions. In these experiments, positron attachment to vibrationally excited molecules is mainly driven by dipole and induced-dipole interactions,^15^,^16^ so the binding energies can be viewed as positron affinities, analogues of electron affinities.

Some of us recently reported on the energy stability of a fundamentally different type of positronic molecule,^17^ formed by two hydride anions and one positron, e^+^[H^–^H^–^]. While the potential energy curves (PECs) undoubtedly pointed out the formation of a molecule, the electron densities around the nuclei were found very similar to those in the isolated atomic species, H^–^ + PsH, where PsH is the same as e^+^[H^–^]. In contrast, the positron density accumulated in the internuclear region, also showing typical signatures of constructive (ground state) and destructive (excited state) interference between atom-centered orbitals, which led to the conclusion that the formation of a positron covalent bond underlies the stabilization of the positron-dihydride compound.

The present study shows that positron bonding is not restricted to the e^+^[H^–^H^–^] molecule. Based on numerical simulations, we provide sufficient evidence of positron covalent bonding in homo- and heteronuclear dihalide anions, e^+^[X^–^Y^–^], with X^–^, Y^–^ = F^–^, Cl^–^, Br^–^. The bonding properties of these positron dihalide anions are compared to those of dialkali cations AB^+^, denoted as e^–^[A^+^B^+^], with A^+^, B^+^ = Na^+^, K^+^, Rb^+^. The dialkali molecules are referred to as purely electronic analogs of the positronic dihalides with isoelectronic atomic cores, *e.g.*, e^+^[F^–^Cl^–^] and e^–^[Na^+^K^+^]. We present a method to calculate the bond energies along with an energy decomposition scheme that provides a clear physical picture of bond formation. Finally, we compare the properties of the positronic dihalides with those previously reported for positronic dihydride.

Since covalent bonds are largely responsible for the structure of matter above the atomic level, the similarities between positron and electronic bonds suggest that the former could give rise to a wide variety of exotic molecular systems. The interest on the interactions between positrons and halide anions dates back to early years of positron and Ps Chemical Physics (for a brief review see Saito^18^) so we revisit those interactions from a new perspective, hopefully expanding the landscapes of the field.

This paper is organized as follows. In Section 2, we summarize the theoretical and computational details of the numerical simulations of positronic and electronic systems. In Section 3, we provide the vibrational parameters, positron and electron densities and binding energies of positronic molecules and contrast them with those of their electronic analogs. In Section 4 we present our concluding remarks and perspectives for future work.

## 2 Methods

Positronic atoms and molecules are described with the any particle molecular orbital (APMO) approach,^19^,^20^ considering electrons and positrons as quantum particles and atomic nuclei as point charges. APMO and other multicomponent approaches have been regularly applied to study positron-containing atoms and molecules.^18^,^21^–^34^ A summary of APMO expressions can be found elsewhere.^20^ In this paper, we label the multicomponent methods after the purely electronic ones, *e.g.*, APMO/HF for Hartree–Fock, APMO/CI for configuration interaction, APMO/MP2 for second-order Møller–Plesset perturbation theory, *etc.*

### 2.1 Energy stability of positronic molecules

Apart from pair annihilation, the following reaction channels are considered to determine the energy stability of the positron dihalides1
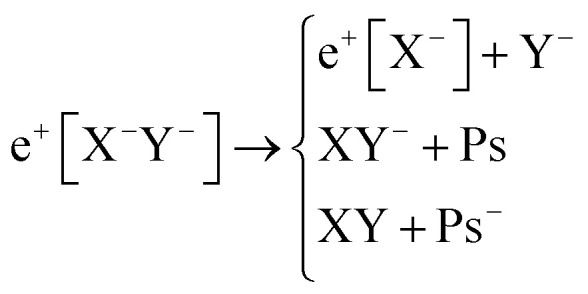



The first decay channel, in which the positron remains bound to the anion with the highest positron affinity (X^–^), has the lowest dissociation energy among the reactions separating X and Y. The second channel involves the formation of a Ps atom and a stable XY^–^ molecular anion, while the third channel leads to the formation of a Ps^–^ anion and a stable XY molecule. The last two channels exhibit the lowest dissociation energies among the reactions producing Ps and Ps^–^, respectively. From top to bottom, the dissociation channels in eqn (1) define the bond energy (BE), the Ps binding energy (PsBE) and the Ps^–^ binding energy (Ps^–^BE) for the positron dihalides.

An accurate description of correlation effects in positronic molecules is a challenging numerical task. To study the energy stability of the e^+^[H^–^H^–^] complex against the dissociation products e^+^[H^–^] + H^–^, it was necessary to resort to the complete basis set limit of high-order CI calculations (up to quadruple excitations, APMO/CISDTQ).^17^ While employing such high-level methods for the positronic dihalides of present interest would be computationally prohibitive, we avoid lower levels of theory, such as APMO/MP2 or APMO/CISD, which could lead to erroneous predictions of the stability of positronic molecules, as was seen for the e^+^[H^–^H^–^] system.

To compromise accuracy and effort, we propose thermodynamic cycles as an alternative method to obtain adequate predictions of the positron dihalides energy stability. From the following cycle,
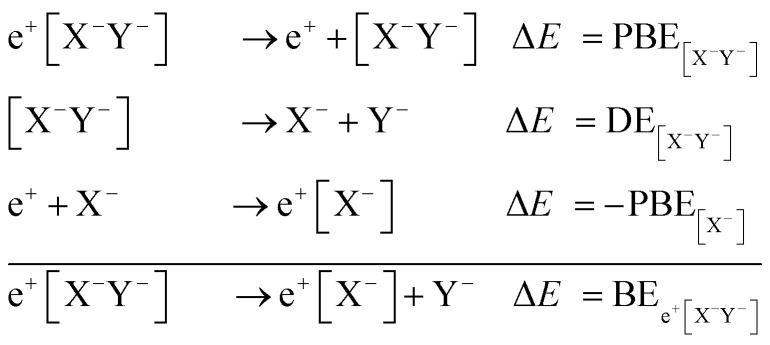


BEs are estimated as,

2 BE_e^+^[X^–^Y^–^]_ = PBE_[X^–^Y^–^]_ + DE_[X^–^Y^–^]_ – PBE_X^–^_.

Similar cycles, presented in the ESI,† are used to estimate PsBEs and Ps^–^BEs as,

3 PsBE_e^+^[X^–^Y^–^]_ = PBE_[X^–^Y^–^]_ + EBE_XY^–^_ + E_Ps_

4 Ps^–^BE_e^+^[X^–^Y^–^]_ = PBE_[X^–^Y^–^]_ + EBE_XY^–^_ + EBE_XY_ + E_Ps^–^_.

The above expressions relate the decay channels of the positronic dihalides, defined in eqn (1), to positron binding energies (PBEs), electron binding energies (EBEs), dissociation energies (DE) of the purely electronic molecular anions ([X^–^Y^–^]), as well as Ps and Ps^–^ ground state energies (E_Ps_ and E_Ps^–^_). In eqn (2)–(4), the DEs, EBEs and PBEs are defined with respect to the equilibrium internuclear distances, except for the unstable [X^–^Y^–^] system, which is assumed to remain at the equilibrium geometry of the e^+^[X^–^Y^–^] complex.

The key aspect of the cycles is the fact that the EBEs, DEs, E_Ps^–^_ and E_Ps_ in eqn (2)–(4) can be calculated with high-level correlated methods, or even taken from the literature, while the best available approximations are employed for the PBEs. In the present study, the EBEs and DEs of the purely electronic systems are evaluated with the coupled cluster method with single, double and perturbative triple excitations (CCSD(T)).^35^ For Ps and Ps^–^ we employ the exact ground state energies (–656 kJ mol^–1^ and –688 kJ mol^–1^,^36^ respectively), while the atomic and molecular PBEs are calculated with the APMO/REN-PP3 propagator method, a renormalized third-order approximation to the diagonal elements of the self energy.^29^ An additional set of BEs, referred to as BE_lb_, is obtained from eqn (2) and the atomic PBEs reported in ref. 18, which were calculated in the full-CI limit of the multi-reference configuration-interaction (MRCI) method. Replacing the APMO/REN-PP3 estimates by the MRCI counterparts serves as a more stringent stability test, since the energies of the positronic dihalides are compared to the best available atomic PBEs. BE_lb_ estimates should thus be viewed as *lower bounds* (the molecular APMO/REN-PP3 PBEs underestimate the electron–positron correlation^29^). The BEs computed only from APMO/REN-PP3 PBEs are expected to be more accurate because the positron–electron correlation is more balanced between the molecular and atomic species.

The energy stability of the purely electron analogs is established in terms of the lowest energy dissociation channel

5 e^–^[A^+^B^+^] → A + B^+^,

where A represents the alkali atom with the highest EBE. Similarly to positronic dihalides, we employ a thermodynamic cycle

6 BE_e^–^[A^+^B^+^]_ = EBE_[A^+^B^+^]_ + DE_[A^+^B^+^]_ – EBE_A^+^_,

to compute the BEs of e^–^[A^+^B^+^] in terms of CCSD(T) EBEs and DEs. The unstable [A^+^B^+^] system is assumed to remain at the equilibrium geometry of the e^–^[A^+^B^+^] complex.

### 2.2 Potential energy curves

The positron dihalides PECs, *E*_e^+^[X^–^Y^–^]_, were computed with the following expression at different internuclear separations *R*,

7 *E*_e^+^[X^–^Y^–^]_(*R*) = *E*_[X^–^Y^–^]_(*R*) – PBE_[X^–^Y^–^]_(*R*).

To obtain PECs consistent with eqn (2) results, we have employed APMO/REN-PP3 estimates of PBE_[X^–^Y^–^]_ and CCSD(T) energies for the purely electronic systems, *E*_[X^–^Y^–^]_.

Ground state PECs were obtained from PBEs calculated as in ref. 29, applying APMO/REN-PP3 self-energy corrections to the occupied positron orbitals generated with APMO/HF calculations for the e^+^[X^–^Y^–^] system. For the first excited state PECs we employed a technique previously used to estimate the excitation energies of radicals as EBE differences between cation virtual orbitals.^37^–^40^ In this scheme, virtual positron orbitals are obtained by including ghost positronic basis functions in APMO/HF calculations for the [X^–^Y^–^] purely electronic system. The APMO/REN-PP3 self-energy corrections are then applied to the second positronic virtual orbital, which allows for estimates of the first excited state PBE of the e^+^[X^–^Y^–^] complex.

The ground state PECs of the purely electronic dialkali molecules are constructed from CCSD(T) total energies, while their first excited state PECs from equation of motion coupled cluster with single and double excitations (EOM-CCSD) excitation energies.^35^ First excited state properties are denoted by the “*” superscript. The bond energy of a stable excited state (BE^*^) is calculated as the difference between energy of the dissociation products and the potential energy minimum.

### 2.3 Energy stability analysis

According to eqn (2)–(4), the energy stability of the positronic complexes can be related to the DEs, EBEs and PBEs. To better understand the mechanisms underlying electronic and positronic bonding, we decompose each of those energy terms as follows.

The DEs of the repulsive [X^–^Y^–^] dianions and [A^+^B^+^] dications are expressed in terms of a modified Coulomb equation that accounts for polarization8
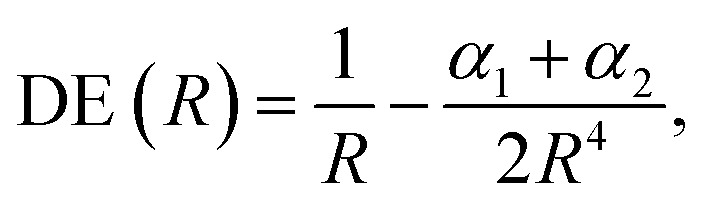
where *R* is the internuclear distance and the *α*'s are the polarizabilities of the ions. The PBEs are decomposed with a scheme previously employed for proton binding energies.^41^ The PBE of e^+^[Z] is decomposed into electrostatic (*E*_el_), relaxation (*E*_rlx_) and correlation (*E*_cor_) contributions. *E*_el_ is defined as the PBE calculated with the frozen electronic density approximation using the Hartree–Fock wave function of Z. *E*_rlx_, which accounts for the electronic density change induced by positron binding, is obtained from the difference between the APMO/HF PBE and *E*_el_. Finally, *E*_cor_ is obtained as the difference of the PBE estimates from a correlated method (APMO/REN-PP3) and APMO/HF. The EBEs are likewise decomposed into the same energy components using CCSD(T) in place of APMO/REN-PP3.

### 2.4 Computational details

A positron basis set with 6s4p3d2f Gaussian-type functions (GTFs) was used in all calculations. The GTF exponents of this basis set, which is referred to as PsX-TZ, were generated by following the Dunning correlation consistent scheme,^42^ as described in the ESI.† Calculations for the e^+^[X^–^] atomic species employed a single basis set centred at the [X^–^] nucleus. For the e^+^[X^–^Y^–^] molecular complexes, three basis sets were used, with expansion centers at the X^–^ and Y^–^ nuclei, and also at their midpoint. The def2-TZVPPD electronic basis set^43^ was employed in all calculations. Ground-state electronic and positronic densities were obtained from CISD and APMO/CISD wave functions calculated at equilibrium distances. In the reported calculations, all electrons and all orbitals were taken into account. Counterpoise corrections were considered in the DE, ΔPBE, ΔEBE and BE calculations to account for the basis set superposition error. The calculations for the positronic systems were carried out with the LOWDIN software,^44^ while those for the purely electronic systems were performed with the ORCA computational package.^45^

## 3 Results and discussion

### 3.1 Positron halides

The PBEs of the e^+^[F^–^], e^+^[Cl^–^] and e^+^[Br^–^] positronic atoms, obtained with the APMO/REN-PP3 method, are shown in Table 1. There is good agreement, within 4–8% (mean error of 35 kJ mol^–1^), with the multi-reference configuration-interaction (MRCI) calculations,^18^ obtained in the full CI limit. The CCSD(T) EBEs of the alkali atoms are within 1–2% of the experimental values (mean error of 7 kJ mol^–1^).^46^ While both the PBEs and EBEs decrease with the ionic core size, the PBEs are always higher comparing the analog systems (*i.e.*, those with isoelectronic ionic cores).

**Table 1:** Positron binding energies (PBE/kJ mol^–1^) of the positronic atoms, e^+^[X^–^], and electron binding energies (EBE/kJ mol^–1^) of the alkali atoms, A

e^+^[X^–^]	APMO/REN-PP3^*a*^	MRCI^*b*^
e^+^[F^–^]	574	600
e^+^[Cl^–^]	497	532
e^+^[Br^–^]	472	516

A	CCSD(T)^*a*^	Exp^*c*^
Na	491	496
K	410	419
Rb	395	403

^*a*^def2-TZVPPD electronic and PsX-TZ positronic basis sets.

^*b*^Multi-reference configuration-interaction (MRCI) results from ref. 18.

^*c*^Experimental results from ref. 46.

### 3.2 Positron dihalides potential energy curves

**Fig. 1:**
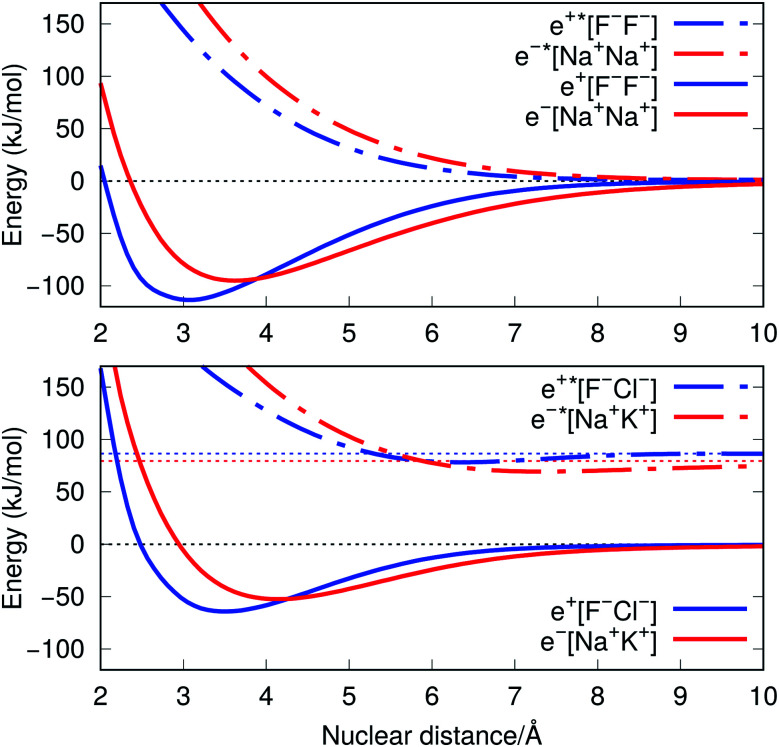
Potential energy curves (PECs) for e^+^[F^–^F^–^], e^–^[Na^+^Na^+^] (top) and e^+^[F^–^Cl^–^], e^–^[Na^+^K^+^] (bottom). The potential curves of the ground and excited states are shown as solid and dot-dashed curves, respectively, and the energies are given with respect to the dissociation products e^+^[F^–^] + F^–^, Na + Na^+^, e^+^[F^–^] + Cl^–^, and Na + K^+^. Horizontal dotted lines indicate the energy of the charge transfer products e^+^[Cl^–^] + F^–^ (blue) and K + Na^+^ (red). PECs were obtained at the CCSD(T), EOM-CCSD and APMO/REN-PP3 (eqn (7)) levels.

The ground-state PECs of the positron complexes e^+^[F^–^F^–^] and e^+^[F^–^Cl^–^], shown in Fig. 1, display potential energy minima which clearly indicate that the addition of a positron to the otherwise repulsive dianions leads to the formation of stable molecular species. Similar stabilization is also found for the other positron dihalides, e^+^[Cl^–^Cl^–^], e^+^[Br^–^Br^–^], e^+^[F^–^Br^–^] and e^+^[Cl^–^Br^–^], as shown in Fig. S1.† Likewise, the ground-state PECs of the purely electronic complexes e^–^[A^+^B^+^] display potential energy minima consistent with the formation of the stable alkali diatomic molecules e^–^[Na^+^Na^+^] and e^–^[Na^+^K^+^]^47^–^49^ (Fig. 1), as well as e^–^[K^+^K^+^], e^–^[Rb^+^Rb^+^], e^–^[Na^+^Rb^+^] and e^–^[K^+^Rb^+^] (Fig. S1†), by the addition of one electron to the repulsive [A^+^B^+^] systems.

Fig. 1 also presents the PECs of first excited states of e^+^[F^–^F^–^], e^–^[Na^+^Na^+^] e^+^[F^–^Cl^–^] and e^–^[Na^+^K^+^]. The PECs of the homonuclear systems (top panel) display repulsive behaviour and their dissociation limits coincide with those of the respective ground states. In contrast, the PECs of heteronuclear systems (bottom panel) present shallow minima and their dissociation limits manifest the energies required to transfer either the positron from F^–^ to Cl^–^, or the electron from Na^+^ to K^+^, as discussed in the next section. Similarly to the cases discussed above, the homonuclear PECs of the remaining positronic and electronic molecules display repulsive behaviour, while the heteronuclear systems exhibit potential energy minima (see Fig. S1†).

The ground-state equilibrium internuclear distances, shown in Table 2, increase with the size of the ionic cores for all systems, while the corresponding force constants decrease. It is worth noting that the positronic systems exhibit shorter bond lengths (0.5–0.8 Å) and larger force constants (3–6 N m^–1^) compared to their purely electronic analogs (isoelectronic ionic cores). The latter results, which suggest more stable positronic bonds, are consistent with the BEs presented in Table 2, obtained from eqn (2) and (6). The BEs of the positronic molecules exceed those of their purely electronic analogs by 4–16 kJ mol^–1^.

**Table 2:** Ground and first excited (denoted by *) state bond distances (*R*/Å), harmonic force constants (*k*/N m^–1^), bond energies (BE/kJ mol^–1^), positron binding energies (PBE/kJ mol^–1^), and electron binding energies (EBE/kJ mol^–1^), for the positronic e^+^[X^–^Y^–^] and electronic e^–^[A^+^B^+^] systems. The differences between molecular and atomic PBEs (ΔPBE/kJ mol^–1^) and EBEs (ΔEBE/kJ mol^–1^), as well as the dissociation energies (DE/kJ mol^–1^), are also given for the [X^–^Y^–^] and [A^+^B^+^] systems

System	*R*	*k*	BE^*a*^	PBE^*b*^	ΔPBE^*b*^	DE^*c*^	System	*R*	*k*	BE^*c*^	EBE^*c*^	ΔEBE^*c*^	DE^*c*^
e^+^[F^–^F^–^]	3.088	15.7	109	1104	528	–419	e^–^[Na^+^Na^+^]	3.623	9.9	93	966	475	–382
e^+^[F^–^Cl^–^]	3.545	10.2	62	998	424	–361	e^–^[Na^+^K^+^]	4.138	6.6	51	875	384	–333
e^+^[F^–^Br^–^]	3.709	8.5	51	969	394	–343	e^–^[Na^+^Rb^+^]	4.319	5.6	42	852	361	–319
e^+^[Cl^–^Cl^–^]	3.869	10.6	83	910	412	–329	e^–^[K^+^K^+^]	4.635	5.6	76	783	373	–297
e^+^[Cl^–^Br^–^]	4.019	9.8	69	883	385	–316	e^–^[K^+^Rb^+^]	4.803	5.1	65	761	351	–286
e^+^[Br^–^Br^–^]	4.149	7.9	78	857	383	–305	e^–^[Rb^+^Rb^+^]	4.972	4.7	68	740	344	–276

System^*c*^	R*	k*	BE*^d^ ^,^^*g*^	PBE*^e^	ΔPBE*^e^ ^,^^*g*^	DE^*c*^	System^*d*^	R*	k*	BE*^f^ ^,^^*g*^	EBE*^f^	ΔEBE*^f^ ^,^^*g*^	DE^*c*^
e^+^* [F^–^Cl^–^]	3.623	9.9	7	699	222	–215	e^–^* [Na^+^K^+^]	7.327	1.0	11	610	201	–189
e^+^* [F^–^Br^–^]	4.138	6.6	10	680	228	–219	e^–^* [Na^+^Rb^+^]	7.258	1.1	12	597	203	–191
e^+^* [Cl^–^Br^–^]	4.319	5.6	7	625	173	–166	e^–^*[K^+^Rb^+^]	9.623	0.3	2	541	146	–144

^*a*^Obtained from eqn (2) with APMO/REN-PP3 PBEs and CCSD(T) DEs.

^*b*^APMO/REN-PP3 ground state calculations.

^*c*^CCSD(T) calculations.

^*d*^Obtained from eqn (2) with APMO/REN-PP3 PBE*s and CCSD(T) DEs.

^*e*^APMO/REN-PP3 first excited state calculations.

^*f*^EOM-CCSD first excited state calculations.

^*g*^Relative to the excited state dissociation products.

The present ground state calculations indicate stable positronic dihalides with respect to the atomic dissociation products described at the same level of theory. A more rigorous check of their energy stability is provided by the BE_lb_ values shown in Table 3, obtained from the most accurate PBEs reported for the atomic fragments.^18^ The BE_lb_ estimates corroborate the stability of the positronic dihalides and provide lower bounds, in view of the more thorough description of positron–electron correlation in the dissociation products (full CI limit of MRCI) than in the molecules (APMO/REN-PP3). The PsBE and Ps^–^BE values reported in Table 3 also point out that dissociation into e^+^[X^–^] + Y^–^ is always the lowest-energy decay channel.

**Table 3:** Lower bounds for the bond energies (BE_lb_), Ps binding energies (PsBE), and Ps^–^ binding energies (Ps^–^BE) for the positronic molecules e^+^[X^–^Y^–^].^*a*^ Energies in kJ mol^–1^

System	BE_lb_	PsBE	Ps^–^BE
e^+^[F^–^F^–^]	85	234	484
e^+^[F^–^Cl^–^]	38	181	361
e^+^[F^–^Br^–^]	27	154	331
e^+^[Cl^–^Cl^–^]	50	144	339
e^+^[Cl^–^Br^–^]	35	123	325
e^+^[Br^–^Br^–^]	36	104	311

^*a*^Obtained from eqn (2)–(4) with APMO/REN-PP3 estimates of PBE_[X^–^Y^–^]_ (Table 2), CCSD(T) estimates of DE and EBE (Tables 2 and S3), MRCI results of PBE_X^–^_ (Table 1)^18^ and the exact energies of E_Ps_ = –656 kJ mol^–1^ and E_Ps^–^_ = –688 kJ mol^–1^.^36^

For completeness, we mention that the excited states of the heteronuclear molecules show similar trends as their ground states. As evident from Table 2, the first excited states of the positronic complexes present shorter bond lengths (1.0–1.4 Å) and stronger force constants (0.7–1.0 N m^–1^) than those of the purely electronic analogs.

For the ground state BEs of all complexes, the basis set superposition error did not exceed 4 kJ mol^–1^, while for the excited states, having larger internuclear separations, the maximum calculated error was 0.4 kJ mol^–1^. Counterpoise corrections are presented in Table S5 in the ESI.†

### 3.3 Positron bond densities and orbitals

**Fig. 2:**
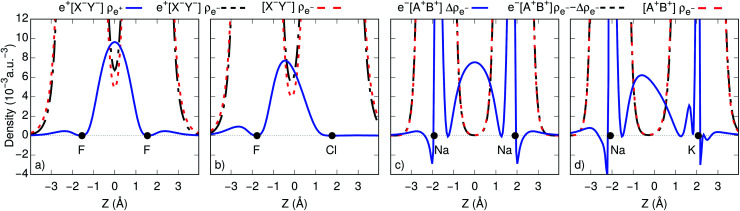
One-dimensional cuts of the positron (*ρ*_e^+^_), electron (*ρ*_e^–^_), and spin (Δ*ρ*_e^–^_) densities for (a) e^+^[F^–^F^–^] and [F^–^F^–^]; (b) e^+^[F^–^Cl^–^] and [F^–^Cl^–^]; (c) e^–^[Na^+^Na^+^] and [Na^+^Na^+^]; (d) e^–^[Na^+^K^+^] and [Na^+^K^+^]. Densities were obtained at the CISD and APMO/CISD levels. The black circles indicate the atomic nuclei.

To gain further insight into bond formation in the ground states, we computed the electron (*ρ*_e^–^_) and positron (*ρ*_e^+^_) densities of the e^+^[X^–^Y^–^] systems, along with the electron and spin (Δ*ρ*_e^–^_) densities of the e^–^[A^+^B^+^] molecules at their respective equilibrium distances. Fig. 2c and d show that the core densities of the [Na^+^Na^+^] and [Na^+^K^+^] dications (red lines) are essentially unchanged by the addition of one electron (black lines). Apart from the vicinity of the nuclei, where the Coulomb attraction gives rise to sharp peaks, the most significant change takes place at the internuclear region. The accumulation of the spin densities (blue lines) manifest the covalent bonding character of the singly occupied molecular orbitals (SOMOs). The remaining purely electronic systems display similar accumulation of spin density at the internuclear region, as shown in Fig. S2,† consistent with the formation of electronic covalent bonds.

The electronic densities of the [F^–^F^–^] and [F^–^Cl^–^] unbound dianions, presented in Fig. 2a and b, also remain virtually unchanged along the internuclear axis after the addition of one positron, except for a slight increase around the density minima between the nuclei. It is therefore evident that the stabilization of the positronic dihalides does not result from electronic bond formation. In contrast, the positron densities (*ρ*_e^+^_) prominently accumulate in the internuclear region of e^+^[F^–^F^–^] and e^+^[F^–^Cl^–^], pointing out the formation of positron covalent bonds.^17^,^33^ The remaining positronic systems also display accumulation of positron densities at the internuclear region along with insignificant change in the electron densities, consistent with the formation of positron bonds (see Fig. S2†).

**Fig. 3:**
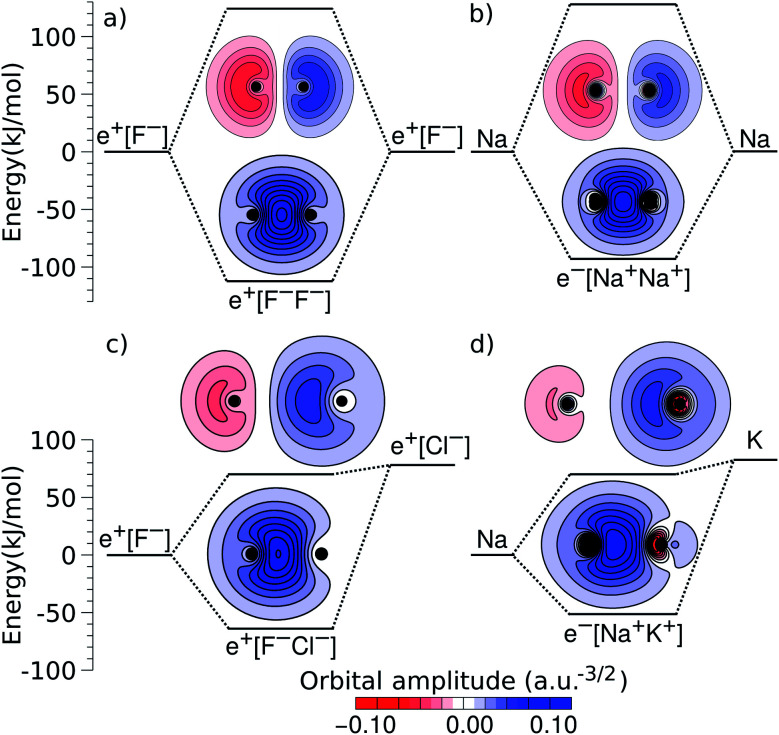
Two-dimensional projections of the singly occupied positronic orbitals of (a) e^+^[F^–^F^–^] and (c) e^+^[F^–^Cl^–^], along with the singly occupied electronic orbitals of (b) e^–^[Na^+^Na^+^] and (d) e^–^[Na^+^K^+^]. In all panels, the lowest unoccupied positronic and electronic orbitals are also shown (the latter can be identified from the nodes and lobes with opposite signs). The horizontal lines indicate the molecular energies and dissociation limits (see Fig. 1). The excited-state energies correspond to vertical transitions for homonuclear complexes and to adiabatic transitions in heteronuclear ones.

An alternative view of covalent bonding is provided by the two-dimensional projections of the singly occupied positron orbitals (SOPOs) in e^+^[F^–^F^–^] and e^+^[F^–^Cl^–^] and the SOMOs in e^–^[Na^+^Na^+^] and e^–^[Na^+^K^+^], presented in Fig. 3. The homonuclear SOPOs and SOMOs display centrosymmetric nodeless distributions in consistency with their σ bonding character. Likewise, heteronuclear SOPOs and SOMOs also display nodeless σ bonding distributions, although distorted towards the anion with higher PBE (SOPO) and the cation with higher EBE (SOMO).

We now turn attention to the excited states. The lowest unoccupied positron orbital (LUPO) of the homonuclear e^+^[F^–^F^–^] molecule, shown in Fig. 3a, exhibits an antisymmetric amplitude with a node at the centre of the molecule, thus manifesting antibonding σ character, and similar features are observed for the lowest unoccupied molecular orbital (LUMO) of the homonuclear e^–^[Na^+^Na^+^] molecule (Fig. 3b). This antibonding character gives rise to the repulsive PECs presented in Fig. 1 and S2.†

In contrast, the LUPO and LUMO of the heteronuclear species are shifted, respectively, towards the anion with the lower PBE (Cl^–^, Fig. 3c), and the cation with the lower EBE (K^+^, Fig. 3d). In view of the large equilibrium distances of the excited states (Table 2), the shifted LUPO (LUMO) amplitudes, and the small energy differences between the LUPOs (LUMOs) and the atomic orbitals of the low-affinity ions, the excited states can be viewed as quasi-atomic states perturbed by the fields generated by the high-affinity ions. The field-induced polarization stabilizes the quasi-atomic states, thus giving rise to the stable molecular excited states. At the infinite internuclear separation limit, the positron (electron) transfers completely to the atomic ion with lower affinity, in consistency with the excited state PECs (Fig. 1).

### 3.4 Positron bonding analysis

To investigate the origins of the stronger bonds in the positronic dihalides, as compared to their purely electronic analogs, we write the BE as the sum of the DE with the difference between the PBE (or EBE) for the molecular and atomic species, *i.e.*, ΔPBE = PBE_[X^–^Y^–^]_ – PBE_[X^–^]_, ΔEBE = EBE_[A^+^B^+^]_ – EBE_[A^+^]_. This decomposition is based on eqn (2) and (6), and the differences ΔPBE and ΔEBE are given in Table 2, along with the BEs.

The DE values presented in Table 2, obtained with the CCSD(T) method, can accurately be fitted as the sum of Coulomb and polarization terms, according to eqn (8) (error ≤ 1%). As observed in Fig. 5, the DEs of the halide anions are lower in magnitude (more negative), at all distances, than those of the isoelectronic alkali cations. According to eqn (8), the lower DEs of halide anions can only arise from their higher polarizabilities (*α*_F_: 11 a.u.^–3^, *α*_Cl_: 30 a.u.^–3^, *α*_Br_: 42 a.u.^–3^), with respect to those of the alkali cations (*α*_Na_: 0.93 a.u.^–3^, *α*_K_: 5.3 a.u.^–3^, *α*_Rb_: 8.6 a.u.^–3^), since the Coulomb repulsion terms are equal. For instance, at the respective equilibrium distances the polarization terms lower the DEs of the dianions by 25–29 kJ mol^–1^, while those of the dications by 1–3 kJ mol^–1^. The polarization contribution to the DEs is therefore partially responsible for the higher BEs and shorter distances of the positronic molecules, compared to their purely electronic analogs.

**Fig. 4:**
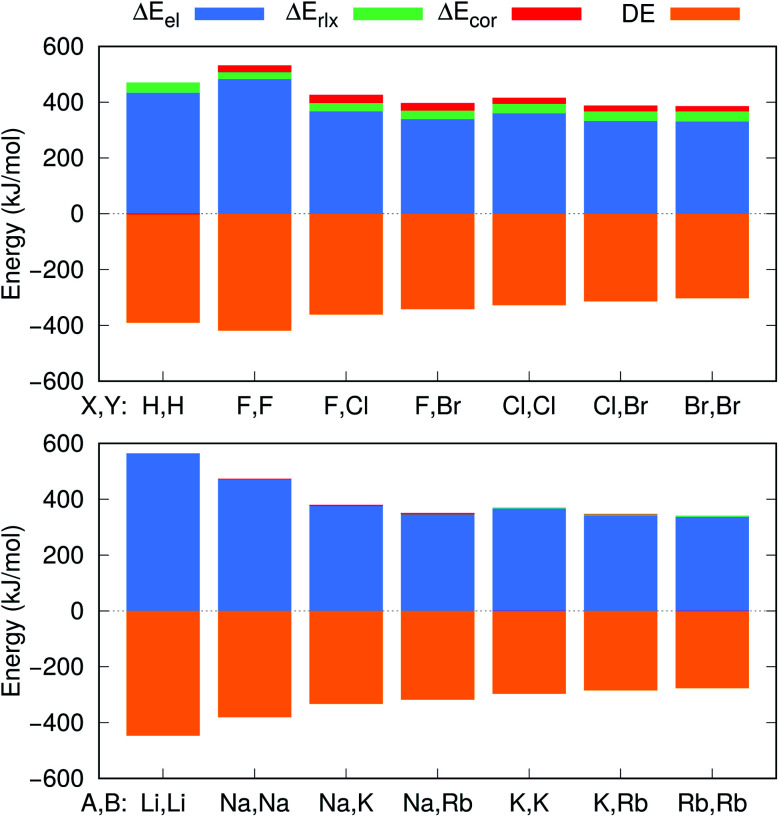
Electrostatic (Δ*E*_el_), relaxation (Δ*E*_rlx_) and correlation (Δ*E*_cor_) contributions to the APMO/REN-PP3 positron binding energy differences between e^+^[X^–^Y^–^] and e^+^[X^–^] (top), and the CCSD(T) electron binding energy differences between e^–^[A^+^B^+^] and A (bottom). The CCSD(T) dissociation energies (DE) for the [X^–^Y^–^] and [A^+^B^+^] systems are also given.

**Fig. 5:**
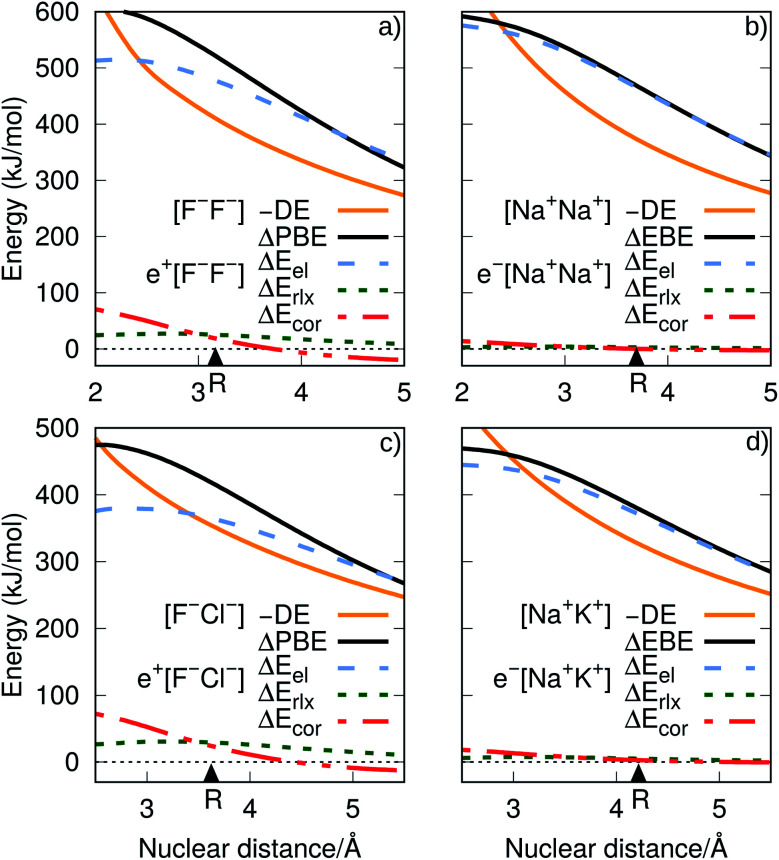
Electrostatic (Δ*E*_el_), relaxation (Δ*E*_rlx_) and correlation (Δ*E*_cor_) contributions to the PBE difference (ΔPBE), as functions of the internuclear distances. Panel (a) corresponds to the difference of e^+^[F^–^F^–^] and e^+^[F^–^], while panel (c) to that of e^+^[F^–^Cl^–^] and e^+^[F^–^]. The decomposition of the EBE differences (ΔEBE) are shown in panel (b) for e^–^[Na^+^Na^+^] and Na, and in panel (d) for e^–^[Na^+^K^+^] and Na. The dissociation energies (DE) of the ionic cores [F^–^F^–^], [F^–^Cl^–^], [Na^+^Na^+^] and [Na^+^K^+^] are presented for comparison. The equilibrium bond distances are indicated by triangles.

The stronger positronic bonds also result from the relatively high positron affinities of the molecular species [X^–^Y^–^] with respect to the atomic products X^–^. As shown in Table 2, the ΔPBEs values exceed by 34–55 kJ mol^–1^ the ΔEBEs values of their purely electronic analogs.

Further insight into the BEs can be gained from the decomposition of the PBEs and EBEs into electrostatic, relaxation and correlation energies, as described in Section 2.3, presented in Fig. 4 and in Table S4.† Analysis of the Δ*E*_el_, Δ*E*_rlx_ and Δ*E*_cor_ contributions for e^+^[X^–^Y^–^] and e^–^[A^+^B^+^] clearly indicates that Δ*E*_el_ accounts for most of the calculated ΔPBE (331–483 kJ mol^–1^) and ΔEBE (336–476 kJ mol^–1^) values. Comparison of the Δ*E*_el_ estimates for the positronic dihalides with their respective dialkali analogs points out modest differences (below 11 kJ mol^–1^) corresponding to less than 3% of the ΔPBE and ΔEBE values. Although the electrostatic contribution is by far the most significant to both ΔPBE and ΔEBE, the relaxation and correlation terms are more important to the positronic species (Δ*E*_rlx_ = 26–36 kJ mol^–1^, Δ*E*_cor_ = 18–27 kJ mol^–1^) than to their purely electronic counterparts (Δ*E*_rlx_ = 1–8 kJ mol^–1^, Δ*E*_cor_ = –1–10 kJ mol^–1^).

It is also instructive to consider in Fig. 5, which shows the dependence of Δ*E*_el_, Δ*E*_rlx_ and Δ*E*_cor_ on the internuclear distance for e^+^[F^–^F^–^], e^–^[Na^+^Na^+^], e^+^[F^–^Cl^–^] and e^–^[Na^+^K^+^]. While Δ*E*_el_ estimates follow the same trend for both positronic and electronic molecules, larger values are found for the electronic dialkalis than for the positronic analogs. More importantly, at the equilibrium distances the Δ*E*_el_ components exceed the DEs (in absolute value) for all systems, pointing out that both positronic and electronic bonds can be attributed to the electrostatic fraction of the PBEs and EBEs. The bonding in positron dihalides and electronic dialkali molecules is therefore essentially an electrostatic process, in which the ionic cores act as effective nuclei with charge ∓1 that interact with a single particle with charge ±1.

Nevertheless, there is also significant contribution from the relaxation and correlation terms to positronic bonding, with a dramatic increase of Δ*E*_cor_ around the equilibrium distances (see Fig. 5). Despite the dominant electrostatic character of the bonding process in all cases, the stronger dihalide positron bonds, as compared to the corresponding dialkali electron bonds, arise partly from the relaxation and correlation effects, and partly from the higher polarizabilities of the ionic cores (see above).

We should remark that only the electronic density of the ionic core is kept frozen in evaluating *E*_el_ term. The positron (spin) density is allowed to relax under the electrostatic potential generated by core density. As a consequence, Δ*E*_el_ accounts for the energetic gain associated with the density accumulation in the internuclear region underlying the formation of positron (electron) covalent bonds. Other energy decomposition analysis (EDAs), such as the Morokuma–Ziegler scheme,^50^ employ different definitions for the electrostatic and relaxation terms. The generalization of purely electronic EDA schemes to multicomponent fermionic systems may be discussed in a future contribution.

We now turn attention to the BE trends with respect to the atomic numbers. For the e^+^[X^–^X^–^] and e^–^[A^+^A^+^] homonuclear complexes, the bonds become less stable as the atomic numbers of X and A increase, as indicated in Table 2. A similar trend is also observed for the molecular PBEs (EBEs) and ΔPBEs (ΔEBEs), as well as the atomic PBEs (EBEs) presented in Table 1. The ionic core charge densities are more localized for the lighter elements, as confirmed by the electrostatic components of the atomic PBEs (EBEs) compiled in Table S4.† We employed a simple model based on molecular orbital theory and the frozen core density approximation, as detailed in the ESI,† to build analytical PECs. From the PBEs (Table 1), we obtained the effective atomic numbers *ζ*_F^–^_ = 0.662, *ζ*_Cl^–^_ = 0.615 and *ζ*_Br^–^_ = 0.600, as well as the respective BEs, 113 kJ mol^–1^, 105 kJ mol^–1^ and 102 kJ mol^–1^. For the electronic complexes, we obtained, from the EBEs, *ζ*_Na^+^_ = 0.611, *ζ*_K^+^_ = 0.559 and *ζ*_Rb^+^_ = 0.549, along with the BE values of 104 kJ mol^–1^, 95 kJ mol^–1^ and 93 kJ mol^–1^, respectively. The model results are consistent with the more sophisticated calculations (Table 2), pointing out a meaningful interpretation of the BE trends in terms of effective core charges.

In contrast, the BEs of the heteronuclear complexes do not show a monotonic dependence on the atomic numbers. Based on molecular orbital theory one could expect the stability of the bonds to increase as the difference between the atomic PBEs (EBEs) decrease. In light of this argument, the trend (PBE_Cl^–^_ – PBE_Br^–^_) = 24 kJ mol^–1^ < (PBE_F^–^_ – PBE_Cl^–^_) = 78 kJ mol^–1^ < (PBE_F^–^_ – PBE_Br^–^_) = 102 kJ mol^–1^ is consistent with the BEs given in Table 2, where BE_e^+^[Cl^–^Br^–^]_ = 72 kJ mol^–1^ > BE_e^+^[F^–^Cl^–^]_ = 64 kJ mol^–1^ > BE_e^+^[F^–^Br^–^]_ = 53 kJ mol^–1^. The same reasoning applies to the atomic EBEs of the purely electronic analogs, where we find the trend (EBE_K^+^_ – EBE_Rb^+^_) = 15 kJ mol^–1^ < (EBE_Na^+^_ – EBE_K^+^_) = 81 kJ mol^–1^ < (EBE_Na^+^_ – EBE_Rb^+^_) = 95 kJ mol^–1^, which is consistent with BE_e^–^[K^+^Rb^+^]_ = 66 kJ mol^–1^ > BE_e^–^[Na^+^K^+^]_ = 52 kJ mol^–1^ > BE_e^–^[Na^+^Rb^+^]_ = 44 kJ mol^–1^. The BEs obtained from the atomic charge simple model presented in the ESI† also follow this trend.

### 3.5 Comparison between positronic dihalides and dihydrides

The positron bond in the e^+^[H^–^H^–^] complex^17^ displays similar features to those of the positron dihalides. For instance, the magnitudes of the e^+^[H^–^H^–^]^17^ equilibrium distance (3.260 Å), force constant (7.4 N m^–1^) and BE (74 kJ mol^–1^) are comparable to their counterparts in Table 2. The e^+^[H^–^H^–^] positron density and orbitals also closely resemble those of the homonuclear dihalides systems. In contrast, a comparison of the e^+^[H^–^H^–^] properties with those of its electronic analog e^–^[Li^+^Li^+^] reveals opposite trends than those discussed above for dihalides and dialkalis. The dihydride positron bond is longer (by 0.154 Å), weaker (by 6.2 N m^–1^) and less energetically stable (by 53 kJ mol^–1^) than the electronic bond in the dilithium cation.

To investigate those differences, Fig. 4 presents the decomposition of ΔPBE and ΔEBE respectively for e^+^[H^–^H^–^] and its purely electronic analog, e^–^[Li^+^Li^+^]. Contrary to the dihalides and dialkalis, where the electrostatic contributions are similar, Δ*E*_el_ is significantly higher (by 129 kJ mol^–1^) in e^–^[Li^+^Li^+^] than in e^+^[H^–^H^–^]. Furthermore, Δ*E*_cor_ is insignificant in e^+^[H^–^H^–^] compared to the Δ*E*_cor_ values of the positron dihalides.

## 4 Conclusions

The present computational study of homo- and hetero-nuclear positron dihalide anions points out that positronic covalent bonding reaches far beyond the e^+^[H^–^H^–^] molecule addressed in a previous study.^17^

To some extent, those positronic molecules are similar to the dialkali cations with isoelectronic atomic cores. The ground state positron and spin densities at the internuclear regions are comparable, as well as the frontier positron and electron orbitals. The vibrational parameters also exhibit similar periodic trends for both positron dihalides and electronic dialkalis. The similarity between the positron complexes and their electronic analogs can be understood from the decomposition of the binding energies, which reveals that the bonding is predominantly electrostatic in all cases. The isoelectronic ionic cores can be viewed as effective nuclei with charge ±1 for the systems with bonding orbitals occupied by a single particle with charge ∓1.

Nevertheless, the positron complexes have shorter bond lengths, stronger force constants and higher bond energies than their purely electronic analogs. The stronger positronic bonds were found to arise partly from the higher polarizabilities of the halide anions, compared to the isoelectronic dialkali cations, and partly from the more significant contributions from relaxation and correlation effects to positronic bonding. The above trends are in contrast with the e^+^[H^–^H^–^] molecule, which has a weaker bond than its electronic analog, e^–^[Li^+^Li^+^].

The present results also raise a number of fascinating questions on new possibilities for positron-bonded systems and the extent of the analogy with the electronic counterparts. Further studies will be conducted in search of molecular anions bound by one positron, intramolecular positron bonds, and even doubly occupied positron orbitals giving rise to covalent bonds of order one.

## Conflicts of interest

There are no conflicts to declare.

## Supplementary Material

Infographic

Supplementary information
