# Supplementary material for: Covalent bonds in positron dihalides
Source: Chem Sci. 2019 Oct 29;11(1):44–52. doi: 10.1039/c9sc04433g (PMC7472554; doi:10.1039/c9sc04433g)

Electronic Supplementary Material (ESI) for Chemical Science.
This journal is © The Royal Society of Chemistry 2019

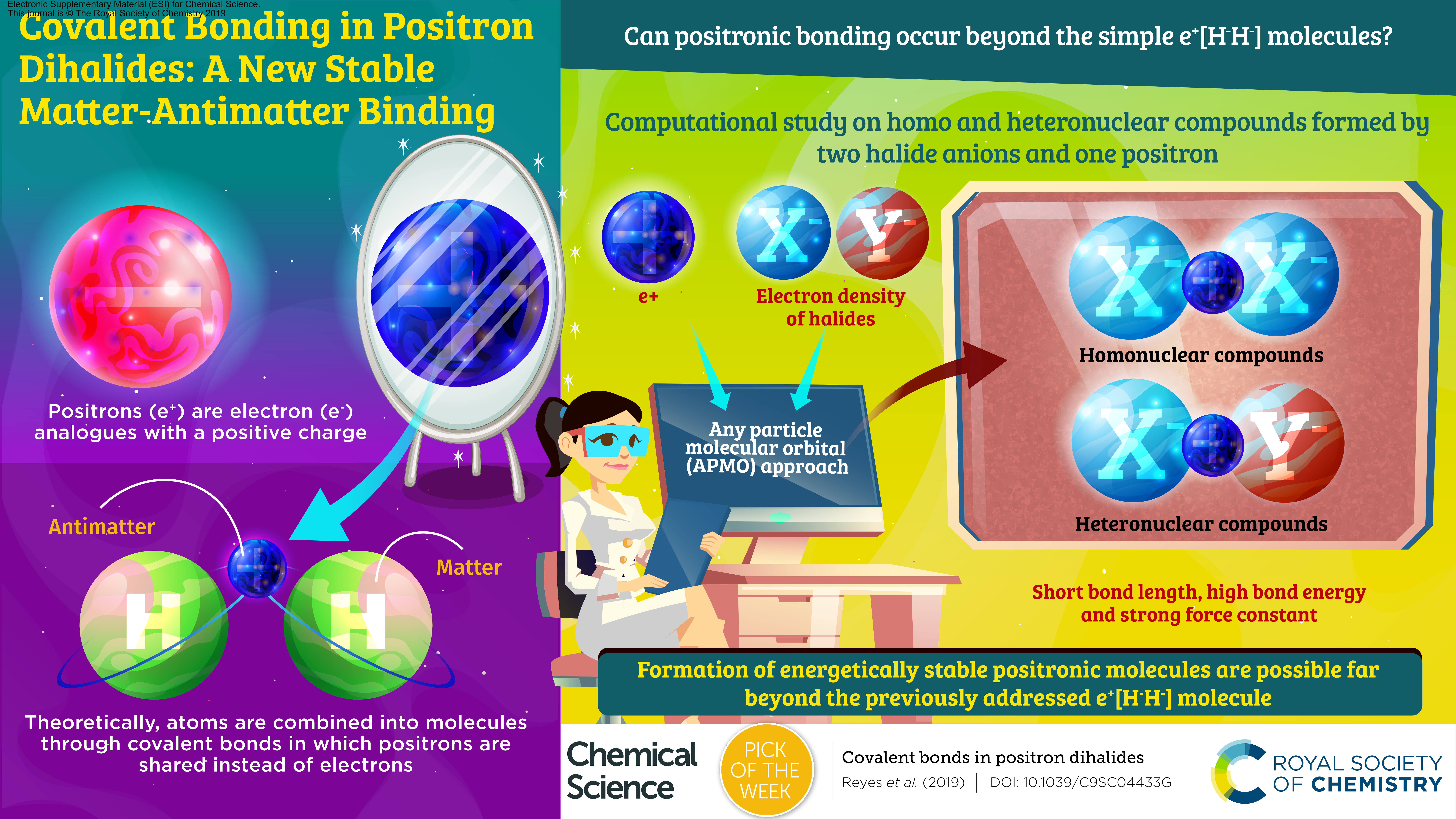

Supplement: Supplementary file 1 [file SC-011-C9SC04433G-s001.pdf]
