# Supplementary material for: Covalent bonds in positron dihalides
Source: Chem Sci. 2019 Oct 29;11(1):44–52. doi: 10.1039/c9sc04433g (PMC7472554; doi:10.1039/c9sc04433g)
Supplement: Supplementary file 2 [file SC-011-C9SC04433G-s002.pdf]

Electronic Supplementary Material (ESI) for Chemical Science.
This journal is © The Royal Society of Chemistry 2019

Covalent bonds in positron dihalides

Supplementary Information

Félix Moncada, Laura Pedraza-González, Jorge Charry, Márcio T. do N. Varella, and Andrés Reyes*

E-mail: areyesv@unal.edu.co

Positron basis sets

Positronic basis sets were generated from uncontracted gaussian-type functions (GTFs). The exponent of the i-th function with angular momentum l, denoted as γ_{il}, was obtained from the even-tempered scheme

$$\gamma_{il} = \alpha_l \beta_l^i \qquad \text{for } i = 0, 1 \ldots n_l - 1 \; . \tag{S.1}$$

Positronic basis sets containing four to seven s-type GTFs were used with the aug-cc-pVQZ electronic basis set for APMO/HF calculations of $e^+[X^-]$ with X=H,F,Cl,Br. The α_l and β_l values for these s-type GTFs were chosen as those that minimize the sum of the APMO/HF total energies for the four systems. We observed that the change in total energy between n_s=6 and n_s=7 was on average 1.1×10^{-5} E_h.

Positronic basis sets containing the previously optimized six s-type GTFs and two to five p-type GTFs were used with the aug-cc-pVQZ electronic basis set for APMO/MP2 calculations of $e^+[X^-]$ with X=H,F,Cl,Br. The α_l and β_l values for these p-type GTFs were chosen as those that minimize the sum of the APMO/MP2 electron-positron correlation

energies for the four systems. We observed that the inclusion of the first two p-type GTFs reduces the correlation energy on average by 0.022 E_h, whereas the change in correlation energy between n_p=4 and n_p=5 was on average 4.7×10^{-5} E_h.

Along those lines, the α_l and β_l values were optimized for two to four d-type GTFs added to positronic basis sets containing 6s- and 5p-type GTFs. We observed that the inclusion of the first two d-type GTFs reduces the correlation energy on average by 0.0042 E_h, whereas the change in correlation energy between n_d=3 and n_d=4 was on average 5.1×10^{-5} E_h.

Similarly, the α_l and β_l values were optimized for two and three f-type GTFs added to positronic basis sets containing 6s-, 5p- and 4d- type GTFs. We observed that the inclusion of the first two f-type GTFs reduces the correlation energy on average 0.0016 E_h and that the addition of the third one reduces this value by 9.1×10^{-5} E_h.

Finally, the α_l and β_l values for two g-type GTFs added to a positronic basis set containing 6s-, 5p-, 4d- and 3f- type GTFs were optimized. The addition of these two GTFs reduces the correlation energy by 3.2×10^{-4} E_h.

Accordingly, we grouped the GTFs with similar contributions to electron-positron correlation energy into the basis sets presented in Table S.1. We refer to these basis sets as PsX-nZ, where $n = \mathrm{D, T, Q}$, indicates double-, triple-, and quadrupole-zeta basis sets, respectively. The optimized parameters α_l and β_l for these positronic basis sets are shown in Table S.1.

The positronic PsX-nZ basis sets show correlation consistent behavior when employed with electronic basis sets of similar quality. In Table S.2 we show the electron-positron correlation energy and PBE for the positronic halides $e^+[X^-]$, calculated with the APMO/MP2 approximation, as a function of the basis set parameter (n). The complete basis set (CBS) extrapolations, E_∞, were obtained from least-squares fits of the expression $E(n) = E_\infty + a/n^{1.88}$.

Potential energy curves and positron and electron densities

Figure S.1 presents the ground state and first excited state potential energy curves (PECs) not shown in the main text: For $e^+[F^-Br^-]$, $e^-[Na^+Rb^+]$, $e^+[Cl^-Cl^-]$, $e^-[K^+K^+]$, $e^+[Cl^-Br^-]$, $e^-[K^+Rb^+]$, $e^+[Br^-Br^-]$ and $e^-[K^+K^+]$. The corresponding positron (ρ_{e^+}), electron (ρ_{e^-}) and spin ($\Delta\rho_{e^-}$) densities are given in Figures S.2 The discussion of these results was addressed in the main text.

Dissociation channels

The lowest energy dissociation channels displayed in equation 1 of the main document were compared to the following processes

$$
\mathrm{e^+[X^-Y^-]} \longrightarrow
\begin{cases}
(1)\ \ \mathbf{e^+[X]^- + Y^-} \\
(2)\ \ \mathbf{XY^- + Ps} \\
(3)\ \ \mathbf{XY + Ps^-} \\
(4)\ \ \mathrm{e^+[Y]^- + X^-} \\
(5)\ \ \mathrm{X^- + Y^- + e^+} \\
(6)\ \ \mathrm{X^- + Y + Ps} \\
(7)\ \ \mathrm{Y^- + X + Ps} \\
(8)\ \ \mathrm{XY + Ps + e^-} \\
(9)\ \ \mathrm{X + Y + Ps^-} \\
(10)\ \ \mathrm{X + Y + Ps + e^-}
\end{cases}
$$

Thermodynamic cycles

Equations 4, 5 and 6 used to calculate PsBE, Ps^{-}BE for the positron complexes and BE of the electron analogs were obtained from the following thermodynamic cycles:

$$
\begin{array}{rll}
e^{+}[X^{-}Y^{-}] & \longrightarrow e^{+} + [X^{-}Y^{-}] & \Delta E \ = \mathrm{PBE}_{[X^{-}Y^{-}]} \\
{[X^{-}Y^{-}]} & \longrightarrow e^{-} + XY^{-} & \Delta E \ = \mathrm{EBE}_{XY^{-}} \\
e^{+} + e^{-} & \longrightarrow \mathrm{Ps} & \Delta E \ = E_{\mathrm{Ps}} \\
\hline
e^{+}[X^{-}Y^{-}] & \longrightarrow \mathrm{Ps} + XY^{-} & \Delta E \ = \mathrm{PsBE}_{e^{+}[X^{-}Y^{-}]}
\end{array}
$$

$$
\begin{array}{rll}
e^{+}[X^{-}Y^{-}] & \longrightarrow e^{+} + [X^{-}Y^{-}] & \Delta E \ = \mathrm{PBE}_{[X^{-}Y^{-}]} \\
{[X^{-}Y^{-}]} & \longrightarrow e^{-} + XY^{-} & \Delta E \ = \mathrm{EBE}_{XY^{-}} \\
XY^{-} & \longrightarrow e^{-} + XY & \Delta E \ = \mathrm{EBE}_{XY} \\
e^{+} + e^{-} + e^{-} & \longrightarrow \mathrm{Ps}^{-} & \Delta E \ = E_{\mathrm{Ps}^{-}} \\
\hline
e^{+}[X^{-}Y^{-}] & \longrightarrow \mathrm{Ps}^{-} + XY & \Delta E \ = \mathrm{Ps}^{-}\mathrm{BE}_{e^{+}[X^{-}Y^{-}]}
\end{array}
$$

$$
\begin{array}{rll}
e^{-}[A^{+}B^{+}] & \longrightarrow e^{-} + [A^{+}B^{+}] & \Delta E \ = \mathrm{EBE}_{[A^{+}B^{+}]} \\
{[A^{+}B^{+}]} & \longrightarrow A^{+} + B^{+} & \Delta E \ = \mathrm{DE}_{[A^{+}B^{+}]} \\
e^{-} + A^{+} & \longrightarrow A & \Delta E \ = -\mathrm{EBE}_{A^{+}} \\
\hline
e^{-}[A^{+}B^{+}] & \longrightarrow e^{-}[A^{+}] + B^{+} & \Delta E \ = \mathrm{BE}_{e^{-}[A^{+}B^{+}]}
\end{array}
$$

PBE, DE and EBE values used for BE calculations are presented in the main document. EBEs used for the PsBE and $PsBE^{-}$ calculations are listed on Table S.3

Energy decomposition

The decomposition of the positron binding energies (PBEs) and electron binding energies (EBEs) is given in Table S.4. In addition to the electrostatic (E_{Elec}), relaxation (E_{Relax}), and correlation (E_{Corr}) contributions, we show the corresponding differences between molecular and atomic species. The discussion of these results was addressed in the main text.

Bond energy dependence on the atomic number

We present a simple model based on molecular orbital theory that allows for a neat interpretation of the bond energy (BE) trend with respect to the atomic numbers of the homonuclear complexes (we employ atomic units to keep the notation simpler). The positron molecular orbitals, Ψ_{XY}, are given by linear combinations of the respective atomic orbitals of X, ψ_x, and Y, ψ_y,

$$\Psi_{\mathrm{XY}} = c_x\psi_x + c_y\psi_y \; . \tag{S.2}$$

Assuming a frozen core density approximation, the positron Hamiltonian is given by

$$\hat{h}_{e^+} = -\frac{\nabla^2}{2} + \hat{V}_x + \hat{V}_y \; , \tag{S.3}$$

where $\hat{V}_x$ and $\hat{V}_x$ are the potential energy operators for the interaction with the X and Y atomic cores, respectively. Defining the integrals

$$S_{xy} = \langle \psi_x \mid \psi_y \rangle \tag{S.4}$$

$$H_{xx} = \langle \psi_x \mid \hat{h}_{e^+} \mid \psi_x \rangle \tag{S.5}$$

$$H_{yy} = \langle \psi_y \mid \hat{h}_{e^+} \mid \psi_y \rangle \tag{S.6}$$

$$H_{xy} = \langle \psi_x \mid \hat{h}_{e^+} \mid \psi_y \rangle \tag{S.7}$$

the energy expectation value is given by

$$\langle E \rangle = \frac{\langle \Psi_{\mathrm{XY}} \mid \hat{h}_{e^+} \mid \Psi_{\mathrm{XY}} \rangle}{\langle \Psi_{\mathrm{XY}} \mid \Psi_{\mathrm{XY}} \rangle} = \frac{c_x^2 H_{xx} + c_y^2 H_{yy} + 2c_x c_y H_{xy}}{c_x^2 + c_y^2 + 2c_x c_y S_{xy}} \; . \tag{S.8}$$

We now suppose that positron atomic orbitals are hydrogenic 1s orbitals produced by the

effective charges of the ion cores ($q_{\text{core}} = -\zeta$, with $\zeta > 0$),

$$\psi_x = \frac{\zeta_x^{3/2}}{\sqrt{\pi}} e^{-\zeta_x |\mathbf{r}-\mathbf{R}_x|} \tag{S.9}$$

$$\psi_y = \frac{\zeta_y^{3/2}}{\sqrt{\pi}} e^{-\zeta_y |\mathbf{r}-\mathbf{R}_y|} \ . \tag{S.10}$$

The effective core charges are obtained from $\zeta = \sqrt{-2\epsilon_{1\text{s}}}$, taking PBE $= -\epsilon_{1\text{s}}$.

To estimate the diagonal matrix elements of the Hamiltonian, we assume that a positron occupying the atomic orbital centered at $\mathbf{R}_x(\mathbf{R}_y)$ interacts with the X(Y) ionic core through the screened potential represented by the effective charge $\zeta_x(\zeta_y)$, although through the unscreened potential with the far lying core Y(X), such that

$$\begin{aligned}
\langle \psi_x \mid \hat{V}_x \mid \psi_x \rangle &= -\zeta_x \langle \psi_x \mid \frac{1}{\mid \mathbf{r}-\mathbf{R}_x \mid} \mid \psi_x \rangle \qquad \langle \psi_y \mid \hat{V}_x \mid \psi_y \rangle = -\langle \psi_y \mid \frac{1}{\mid \mathbf{r}-\mathbf{R}_x \mid} \mid \psi_y \rangle \\
\langle \psi_y \mid \hat{V}_y \mid \psi_y \rangle &= -\zeta_y \langle \psi_y \mid \frac{1}{\mid \mathbf{r}-\mathbf{R}_y \mid} \mid \psi_x \rangle \qquad \langle \psi_x \mid \hat{V}_y \mid \psi_x \rangle = -\langle \psi_x \mid \frac{1}{\mid \mathbf{r}-\mathbf{R}_y \mid} \mid \psi_x \rangle
\end{aligned} \tag{S.11}$$

For the off-diagonal elements, arising from the superposition of the orbital amplitudes centered on both ionic cores, we consider the average of the screened and unscreened interactions,

$$\begin{aligned}
\langle \psi_x \mid \hat{V}_x \mid \psi_y \rangle = \langle \psi_y \mid \hat{V}_x \mid \psi_x \rangle &= -\frac{1+\zeta_x}{2} \langle \psi_y \mid \frac{1}{\mid \mathbf{r}-\mathbf{R}_x \mid} \mid \psi_x \rangle \\
\langle \psi_x \mid \hat{V}_y \mid \psi_y \rangle = \langle \psi_y \mid \hat{V}_y \mid \psi_x \rangle &= -\frac{1+\zeta_y}{2} \langle \psi_y \mid \frac{1}{\mid \mathbf{r}-\mathbf{R}_y \mid} \mid \psi_x \rangle
\end{aligned} \tag{S.12}$$

For the homonuclear complexes, $H_{xx} = H_{yy}$ and $\zeta_x = \zeta_y = \zeta$. The solution of the secular equation S.8 leads to the well known result,

$$\text{E} = \frac{H_{xx} \pm H_{xy}}{1 \pm S_{xy}} \ , \tag{S.13}$$

where the positive root corresponds to the ground (bonding) state, and the matrix elements

are given by

$$S_{xy} = \left(1 + \zeta R + \frac{1}{3}\zeta^2 R^2\right) e^{-\zeta R} \tag{S.14}$$

$$H_{xx} = -\frac{\zeta^2}{2} - \frac{1}{R} + e^{-2\zeta R}\left(\zeta + \frac{1}{R}\right) \tag{S.15}$$

$$H_{xy} = \frac{\zeta^2}{2}\left(1 + \zeta R - \frac{\zeta^2 R^2}{3}\right) e^{-\zeta R} - \frac{\zeta}{2}(\zeta + 1)(\zeta R + 1)e^{-\zeta R} \tag{S.16}$$

The solution of eq. S.8, can also be obtained for the heteronuclear case,

$$\begin{aligned} \mathrm{E} &= \frac{-b \pm \sqrt{b^2 - 4ac}}{2a} \\ a &= 1 - S_{xy}^2 \\ b &= -H_{xx} + H_{xy}S_{xy} + H_{yx}S_{xy} - H_{yy} \\ c &= H_{xx}H_{yy} - H_{xy}H_{yx} \end{aligned} \tag{S.17}$$

where the negative root corresponds to the ground state, and the matrix elements can be written as

$$S_{xy} = \frac{8\zeta_x^{3/2}\zeta_y^{3/2}e^{-R(\zeta_x+\zeta_y)}}{R\left(\zeta_x^2 - \zeta_y^2\right)^3}\left(e^{\zeta_y R}(4\zeta_x\zeta_y + \zeta_x^2\zeta_y R - \zeta_y^3 R) - e^{\zeta_x R}(4\zeta_x\zeta_y + \zeta_x\zeta_y^2 R - \zeta_x^3 R)\right) \tag{S.18}$$

$$H_{xx} = -\frac{\zeta_x^2}{2} - \frac{1}{R} + e^{-2\zeta_x R}\left(\zeta_x + \frac{1}{R}\right) \tag{S.19}$$

$$H_{yy} = -\frac{\zeta_y^2}{2} - \frac{1}{R} + e^{-2\zeta_y R}\left(\zeta_y + \frac{1}{R}\right) \tag{S.20}$$

$$\begin{aligned} H_{xy} =& \frac{2\zeta_x^{3/2}\zeta_y^{3/2}e^{-R(\zeta_x+\zeta_y)}}{R\left(\zeta_x^2 - \zeta_y^2\right)^3}\Big[Re^{\zeta_x R}\left(\zeta_x\zeta_y^4 - \zeta_x^5\right) - Re^{\zeta_y R}\left(\zeta_x^4\zeta_y - \zeta_y^5\right) \\ &+ \left(e^{\zeta_x R} - e^{\zeta_y R}\right)\left(4\zeta_x^3\zeta_y + 4\zeta_x\zeta_y^3 - 2\zeta_x^3 - 2\zeta_y^3 + 2\zeta_x^2\zeta_y + 2\zeta_x\zeta_y^2 - (\zeta_x^2 - \zeta_y^2)R\right)\Big]. \end{aligned} \tag{S.21}$$

To generate analytical potential energy curves, we consider an unscreened $1/R$ term for the core-core repulsion, since the overlap between the core densities is assumed negligible around and above the equilibrium distance.

In case we approximate the atomic 1s energies by the electrostatic components, E_{el}, we obtain the effective core charges $\zeta_F = 0.603$, $\zeta_{Cl} = 0.532$ and $\zeta_{Br} = 0.514$, from which we estimate the BEs, respectively, as 103 kJ/mol, 90 kJ/mol and 87 kJ/mol. It is clear that the BE values increase with the effective charge reflecting the localization of the core densities, thus predicting the trend with respect to the atomic numbers. In case we approximate the 1s atomic energies by the PBEs (which also take the relaxation and correlation contributions into account), the effective charges become $\zeta_F = 0.662$, $\zeta_{Cl} = 0.615$ and $\zeta_{Br} = 0.600$, and the respective BEs, 113 kJ/mol, 105 kJ/mol and 102 kJ/mol, such that the trend is once more predicted. The agreement of the model with the BEs given in Table 2 is better for the $e^+[F^-F^-]$ complex, as expected from its more compact core density and the less significant contribution from correlation.

The atomic charge model also predicts the trend for the purely electronic species. From the effective charges $\zeta_{Na} = 0.611$, $\zeta_K = 0.559$ and $\zeta_{Rb} = 0.549$, estimated from the atomic EBEs, we obtain the BE values of 104 kJ/mol, 95 kJ/mol and 93 kJ/mol, respectively. Since the EBEs are essentially given by the electrostatic components ($> 90\%$), taking into account the relaxation and correlation components has little impact on the effective charges ($< 5\%$).

For the heteronuclear complexes, the atomic charge model with the effective charges obtained from the PBE values, predicts the following trend: $BE_{e^+[Cl^-Br^-]}$=92 kJ/mol> $BE_{e^+[F^-Cl^-]}$=74 kJ/mol> $BE_{e^+[F^-Br^-]}$=64 kJ/mol. For the electron analogs, a similar trend is observed: $BE_{e^-[K^+Rb^+]}$=82 kJ/mol> $BE_{e^-[Na^+K^+]}$=65 kJ/mol> $BE_{e^-[Na^+Rb^+]}$=59 kJ/mol. These are the same trends observed for the BE values presented in Table 2, which reveals that complexes with a large difference between their atomic PBEs (EBEs) have weaker bonds.

Table S.1: Even-tempered parameters for the PsX-DZ, PsX-TZ and PsX-QZ positron basis sets[a].

Basis	l	s	p	d	f	g
PsX-DZ	n_l	5	3	2		
	α_l	0.0190	0.0591	0.1165		
	β_l	2.734	2.729	2.685		
PsX-TZ	n_l	6	4	3	2	
	α_l	0.0171	0.0496	0.0951	0.1596	
	β_l	2.545	2.503	2.458	2.678	
PsX-QZ	n_l	7	5	4	3	2
	α_l	0.0160	0.0417	0.0813	0.1177	0.1419
	β_l	2.424	2.255	2.224	2.412	2.711

[a] α_l values in a.u.$^{-2}$

Table S.2: APMO/MP2 electron-positron correlation (E(2)[e^-e^+]) and positron binding energies (PBE) for monoatomic $e^+[X^-]$ systems employing different combinations of electron and positron basis sets. All energies in E_h.

	n	2	3	4	∞
	e^- basis set	aug-cc-pVDZ	aug-cc-pVTZ	aug-cc-pVQZ	CBS[a]
	e^+ basis set	PsX-DZ	PsX-TZ	PsX-QZ	
$e^+[F^-]$	E(2)[e^-e^+]	-0.0201	-0.0251	-0.0271	-0.0297
	PBE	0.1974	0.2029	0.2052	0.2079
$e^+[Cl^-]$	E(2)[e^-e^+]	-0.0258	-0.0314	-0.0335	-0.0363
	PBE	0.1670	0.1730	0.1754	0.1785
$e^+[Br^-]$	E(2)[e^-e^+]	-0.0256	-0.0317	-0.0339	-0.0370
	PBE	0.1575	0.1641	0.1666	0.1699

[a] Complete basis set extrapolation obtained from $E(n) = E_\infty + a/n^{1.88}$ ($R^2 > 0.999$).

Table S.3: CCSD(T) Adiabatic electron binding energies (EBE) for dihalides monoanions, XY^-, and dianions, $[X^-Y^-]$. All energies in kJ/mol.

System	EBE[a]	System	EBE
$[F^-F^-]$	-214	FF^-	282
$[F^-Cl^-]$	-161	FCl^-	211
$[F^-Br^-]$	-159	FBr^-	209
$[Cl^-Cl^-]$	-111	$ClCl^-$	227
$[Cl^-Br^-]$	-104	$ClBr^-$	233
$[Br^-Br^-]$	-96	$BrBr^-$	239

[a] The unstable $[X^-Y^-]$ systems were calculated at the equilibrium distance of the positron complexes $e^+[X^-Y^-]$.

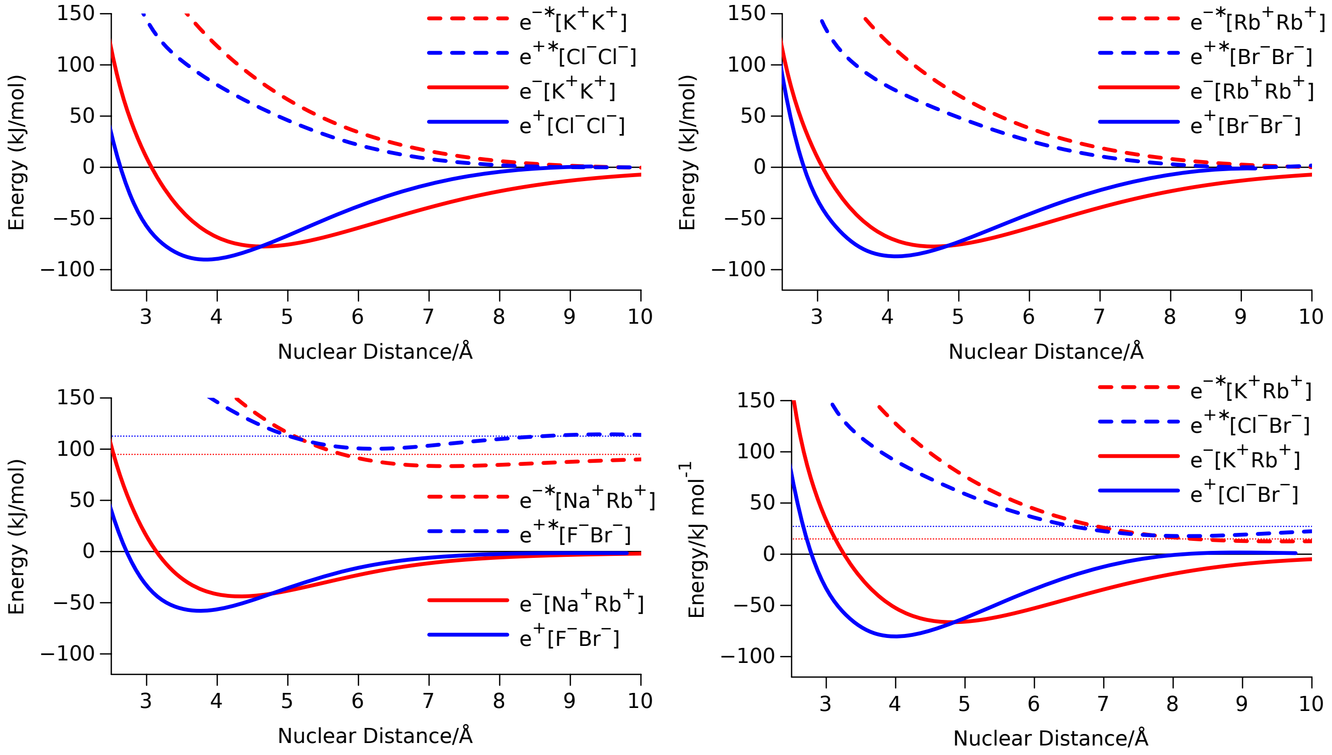

Figure S.1: Potential energy curves (PECs) for $e^+[Cl^-Cl^-]$ and $e^-[K^+K^+]$ (top,left), $e^+[Br^-Br^-]$ and $e^-[Rb^+Rb^+]$ (top,right), $e^+[F^-Br^-]$ and $e^-[Na^+Rb^+]$ (bottom,left), $e^+[Cl^-Br^-]$ and $e^-[K^+Rb^+]$ (bottom,right). Solid lines represent ground states and dashed lines first excited states. Energies given with respect to the dissociation products $e^+[X^-]+Y^-$ and $A+B^+$ where X(A) is the halide(alkali) with the largest positron(electron) affinity. Horizontal dotted lines represent the energy of the charge transfer products $e^+[Y^-]+X^-$ (blue) and $B+A^+$ (red). PECs were obtained at the CCSD(T), EOM-CCSD and APMO/REN-PP3 levels.

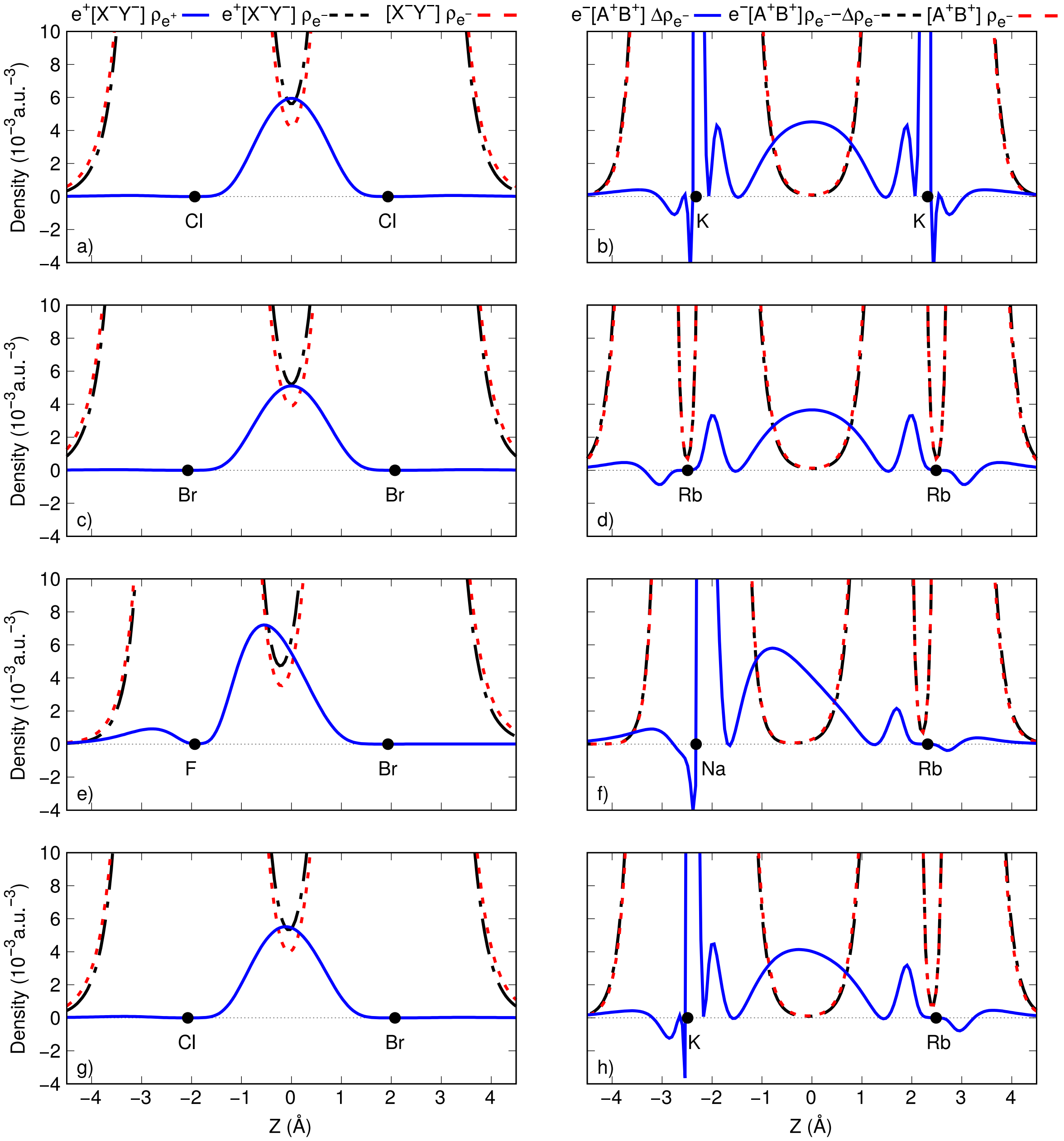

Figure S.2: One-dimensional cuts of the positron (ρ_{e^+}), electron (ρ_{e^-}), and spin ($\Delta\rho_{e^-}$) densities for (a) $e^+[Cl^-Cl^-]$ and $[Cl^-Cl^-]$; (b) $e^-[K^+K^+]$ and $[K^+K^+]$; (c) $e^+[Br^-Br^-]$ and $[Br^-Br^-]$; (d) $e^-[Rb^+Rb^+]$ and $[Rb^+Rb^+]$; (e) $e^+[F^-Br^-]$ and $[F^-Br^-]$; (f) $e^-[Na^+Rb^+]$ and $[Na^+Rb^+]$; (g) $e^+[Cl^-Br^-]$ and $[Cl^-Br^-]$; (h) $e^-[K^+Rb^+]$ and $[K^+Rb^+]$. Densities were obtained at the CISD and APMO/CISD levels. Black dots depict point charge nuclei

Table S.4: Electrostatic (E_{Elec}), relaxation (E_{Relax}) and correlation (E_{Corr}) contributions to the positron binding energies (PBEs) of $e^+[X^-Y^-]$ and $e^+[X^-]$ and the electron binding energies (EBEs) of $e^-[A^+B^+]$ and A. Energies in kJ/mol

System	$e^+[X^-Y^-]$			$e^+[X^-]$			$e^+[X^-Y^-] - e^+[X^-]$		
X,Y	E_{Elec}	E_{Relax}	E_{Corr}	E_{Elec}	E_{Relax}	E_{Corr}	ΔE_{Elec}	ΔE_{Relax}	ΔE_{Corr}
F,F	961 (87%)	30 (3%)	112 (10%)				483 (91%)	26 (5%)	21 (4%)
F,Cl	846 (85%)	34 (3%)	118 (12%)	478 (83%)	5 (1%)	92 (16%)	368 (87%)	30 (7%)	27 (6%)
F,Br	818 (84%)	35 (4%)	116 (12%)				340 (86%)	31 (8%)	25 (6%)
Cl,Cl	733 (81%)	38 (4%)	140 (15%)	372 (75%)	3 (1%)	121 (24%)	361 (87%)	34 (8%)	19 (5%)
Cl,Br	705 (80%)	38 (4%)	140 (16%)				333 (86%)	35 (9%)	18 (5%)
Br,Br	678 (79%)	39 (5%)	140 (16%)	347 (73%)	3 (1%)	122 (26%)	331 (86%)	36 (9%)	18 (5%)
System	$e^-[A^+B^+]$			A			$e^-[A^+B^+]- A$		
A,B	E_{Elec}	E_{Relax}	E_{Corr}	E_{Elec}	E_{Relax}	E_{Corr}	ΔE_{Elec}	ΔE_{Relax}	ΔE_{Corr}
Na,Na	948 (98%)	3 (0%)	15 (2%)				471 (99%)	3 (1%)	1 (0%)
Na,K	852 (97%)	6 (1%)	18 (2%)	476 (97%)	1 (0%)	14 (3%)	375 (98%)	5 (1%)	4 (1%)
Na,Rb	821 (96%)	6 (1%)	24 (3%)				345 (96%)	6 (2%)	10 (3%)
K,K	753 (96%)	8 (1%)	22 (3%)	386 (94%)	1 (0%)	23 (6%)	367 (98%)	7 (2%)	-1 (-0%)
K,Rb	728 (96%)	9 (1%)	25 (3%)				342 (97%)	8 (2%)	1 (0%)
Rb,Rb	703 (95%)	9 (1%)	27 (4%)	366 (93%)	1 (0%)	28 (7%)	337 (98%)	8 (2%)	-1 (-0%)

E_{Elec} is the PBE/EBE calculated with the frozen electronic orbitals of $[X^-Y^-]/[A+B^+]$

E_{Relax} is the difference between the PBE/EBE calculated at the HF level and E_{Elec}

E_{Corr} is the difference between the PBE/EBE calculated with the correlated method (APMO/REN-PP3 or CCSD(T)) and HF.

Table S.5: Counterpoise corrections to the Bond Energy (BE) of $e^+[X^-Y^-]$ and $e^-[A^+B^+]$, to the molecular-atomic PBE and EBE differences of $e^+[X^-Y^-]$-$e^+[X^-]$ and $e^-[A^+B^+]$-A, and the dissociation energies (DE) of $[X^-Y^-]$ and $[A^+B^+]$. Energies in kJ/mol

System	BE	ΔPBE	DE
$e^+[F^-F^-]$	1.4	0.8	0.5
$e^+[F^-Cl^-]$	1.3	0.7	0.6
$e^+[F^-Br^-]$	1.5	0.6	0.8
$e^+[Cl^-Cl^-]$	2.5	1.4	1.1
$e^+[Cl^-Br^-]$	2.6	1.3	1.3
$e^+[Br^-Br^-]$	3.4	1.8	1.6
$e^{+*}[F^-Cl^-]$	0.2	0.2	0.0
$e^{+*}[F^-Br^-]$	0.4	0.4	0.1
$e^{+*}[Cl^-Br^-]$	0.2	0.2	0.0
System	BE	ΔEBE	DE
$e^-[Na^+Na^+]$	1.7	0.2	1.5
$e^-[Na^+K^+]$	1.1	0.2	0.9
$e^-[Na^+Rb^+]$	1.2	0.2	1.0
$e^-[K^+K^+]$	1.1	0.2	0.9
$e^-[K^+Rb^+]$	1.3	0.2	1.1
$e^-[Rb^+Rb^+]$	1.6	0.2	1.4
$e^{-*}[Na^+K^+]$	0.2	0.0	0.1
$e^{-*}[Na^+Rb^+]$	0.3	0.0	0.2
$e^{-*}[K^+Rb^+]$	0.1	0.0	0.1
